# Dissemination and Implementation of a Social Group Intervention for Older Rural Veterans With PTSD

**DOI:** 10.1093/geroni/igaf122.630

**Published:** 2025-12-31

**Authors:** Anica Pless Kaiser, Jennifer Daks, Jay Gregg, Aaron Stusser, Annika Goldman, Shawn Dunlap, Madison Strouse, Jennifer Moye

**Affiliations:** VA Boston Health Care System, Boston, Massachusetts, United States; VA Boston Health Care System, Boston, Massachusetts, United States; Durham VA Medical Center, Durham, North Carolina, United States; Montana VA Health Care System, Fort Harrison, Montana, United States; Durham VA Medical Center, Durham, North Carolina, United States; VA Bedford Health Care System, Bedford, Massachusetts, United States; VA Bronx Healthcare System, New York, New York, United States; VA Boston Health Care System, Boston, Massachusetts, United States

## Abstract

Older rural Veterans confront greater social isolation and health comorbidities, and have lower access to healthcare than Veterans in urban areas. Additionally, older rural Veterans with posttraumatic stress disorder (PTSD) are at elevated risk for social isolation, loneliness, and low social support. The purpose of this VA Office of Rural Health funded project was to disseminate and evaluate an 8-9 week group intervention “Enhancing Social Function for Older Veterans with PTSD” (ESVP), delivered in-person or virtually. Dissemination proceeded in several steps. (1) We identified potentially interested clinicians via 10-minute presentations at regional VA Geriatric Mental Health Champion-led meetings. (2) We provided 60-minute trainings to six clinicians, offering co-facilitation and consultation. (3) We partnered with behavioral health clinicians serving older rural Veterans at two sites. At the Montana site, our team co-facilitated two virtual ESVP groups (N = 9 Veterans) with Veterans from Montana, South Dakota, and Massachusetts. At the North Carolina site, clinicians co-facilitated one in-person ESVP group (N = 4 Veterans). (4) Leaders provided week by week feedback during consultation calls. Across the three groups, average session attendance was high (79%), despite initial technological difficulties in the virtual groups. Veterans reported modest gains in social function and decreased depressive symptoms pre to post group and provided positive qualitative feedback and suggestions for improvement. Clinicians reported high applicability (M = 4.9/5), feasibility (M = 4.4/5), and helpfulness (M = 4.8/5) of ESVP, and provided valuable feedback to inform future efforts to disseminate ESVP to older Veterans in rural areas.

